# Increased adipose tissue expression of IL‐18R and its ligand IL‐18 associates with inflammation and insulin resistance in obesity

**DOI:** 10.1002/iid3.170

**Published:** 2017-05-15

**Authors:** Rasheed Ahmad, Reeby Thomas, Shihab Kochumon, Sardar Sindhu

**Affiliations:** ^1^ Immunology and Innovative Cell Therapy Unit Dasman Diabetes Institute (DDI) Dasman Kuwait

**Keywords:** Adipose tissue, IL‐18, IL‐18R, inflammation, insulin resistance, obesity, type‐2 diabetes

## Abstract

**Introduction:**

The proinflammatory cytokine IL‐18 is involved in the pathogenesis of metabolic syndrome. While the changes in IL‐18 are known, IL‐18R expression and relationship with IL‐18 and other inflammatory markers in the adipose tissue in obesity/type‐2 diabetes (T2D) remain unclear.

**Methods:**

We, therefore, determined the adipose tissue expression of IL‐18R and IL‐18 mRNA/protein in lean, overweight, and obese individuals with and without T2D, 15 each, using qRT‐PCR, immunohistochemistry, and confocal microscopy. Data (mean ± SEM) were analyzed using unpaired *t*‐test and Pearson's correlation (*r*); all *P* values ≤0.05 were considered statistically significant.

**Results:**

We found the upregulated gene/protein expression of IL‐18R and IL‐18 in non‐diabetic obese/overweight as compared with lean individuals (*P* < 0.05). BMI correlated positively (*P* < 0.05) with the adipose tissue expression of IL‐18R (mRNA: *r* = 0.90 protein: *r* = 0.84) and IL‐18 (mRNA: *r* = 0.84 protein: *r* = 0.80). Similarly, in T2D individuals, gene and protein expression of IL‐18R/IL‐18 was significantly higher in obese as compared with overweight/lean individuals. The BMI was associated with the changes in both IL‐18R (mRNA: *r* = 0.55 protein: *r* = 0.50) and IL‐18 (mRNA: *r* = 0.53 protein: *r* = 0.57) expression. IL‐18R/IL‐18 gene expression in the adipose tissue was positively associated (*P* < 0.05) with local gene expression of other inflammatory markers including CD11c, CD86, CD68, CD163, TNF‐α, and CCL5. Homeostatic model assessment of insulin resistance (HOMA‐IR) was higher in diabetic/non‐diabetic obese and it correlated with BMI (*P* < 0.05). IL‐18R and IL‐18 mRNA/protein expression in obesity was associated with HOMA‐IR only in non‐diabetics.

**Conclusions:**

The adipose tissue IL‐18R/IL‐18 expression is enhanced in obesity which associates with proinflammatory gene signature and insulin resistance in these individuals.

## Introduction

Obesity is the leading cause of chronic low grade inflammation which results in insulin resistance and development of type‐2 diabetes (T2D) and its associated metabolic complications. The changes in the expression of proinflammatory cytokines and the increased numbers of various inflammatory cell populations in the expanding adipose tissue play a critical role in the induction of metabolic inflammation. The most common cytokines/chemokines that are associated with metabolic inflammation include tumor necrosis factor (TNF)‐α, interleukin (IL)‐1β, IL‐6, interferon (IFN)‐γ, C‐C chemokine ligand (CCL)‐2 or macrophage chemoattractant protein (MCP)‐1, C‐X‐C chemokine ligand (CXCL)‐8/IL‐8, and CCL‐5 or regulated on activation normal T‐cell expressed and secreted (RANTES) 1. IL‐18, also called IFNγ‐inducing factor, is a unique proinflammatory cytokine that belongs to the IL‐1 family of cytokines and is constitutively expressed and secreted by monocytes/macrophages, Kupffer cells, dendritic cells, as well as other cell types such as epithelial/endothelial cells, and vascular smooth muscle cells 2. IL‐18 is an 18 kDa glycoprotein derived by caspase‐1‐mediated cleavage of its 23 kDa precursor called pro‐IL18 3. IL‐18 plays immunoregulatory role via the induction of IFN‐γ which exerts changes in a variety of cell types and is associated with the pathogenesis of several inflammatory diseases such as rheumatoid arthritis, Crohn's disease, and T2D. In regard with metabolic syndrome, IL‐18 is associated with obesity, insulin resistance, hypertension, atherosclerotic lesions, dyslipidemia, and cardiovascular disease 4.

IL‐18 mediates its bioactivities through a heterodimeric IL‐18 receptor (IL‐18R) comprising of α‐ and β‐chains that are expressed on a wide variety of cells including macrophages, naive T/B lymphocytes, natural killer (NK) cells, neutrophils, endothelial cells, smooth muscle cells, and chondrocytes 5. IL‐18Rα binds IL‐18 with low affinity while IL‐18Rβ does not bind the ligand directly but rather binds with high affinity to IL‐18/IL‐18Rα complex and induces intracellular signal transduction including the recruitment and activation of myeloid differentiation factor 88 (MyD88) and IL‐1R‐associated kinase (IRAK) to the receptor complex 6, 7. The downstream activation promotes the maturation of T‐lymphocytes and natural killer cells as well as the expression of IFN‐γ, other proinflammatory cytokines, chemokines, cell adhesion molecules, and matrix metalloproteinases 8, 9.

Whereas, IL‐18 expression in metabolic disease was investigated 10, 11, 12, modulations in the expression of IL‐18R with regard to IL‐18 and other inflammatory markers in the adipose tissue in obesity and/or T2D still remain unclear. Therefore, in this study, to further extend the knowledge and highlight importance in metabolic inflammation, we determined simultaneous changes in the receptor and its ligand and, herein, our data show upregulated adipose tissue expression of IL‐18R and IL‐18 in obese diabetic/non‐diabetic individuals. This altered expression was found to be associated positively with monocyte/macrophage markers and expression of TNF‐α and CCL‐5. These changes also correlated with homeostatic model assessment of insulin resistance (HOMA‐IR) index.

## Materials and Methods

### Study population

A total of 15 T2D (eight male and seven female, aged 45–64 years) and 15 non‐diabetic (four male and 11 female, aged 24–60 years) individuals were recruited in the study through the clinics of Dasman Diabetes Institute (DDI), Kuwait. Those of age <18 years or with serious comorbid conditions including diseases of the lung, kidney, heart, and liver, or those with hematologic or immune disorders, pregnancy, type‐1 diabetes, or malignancy were excluded. The participants were subclassified as lean, overweight, and obese based on body mass index (BMI). The T2D group comprised of one lean (BMI = 23.54 kg/m^2^); seven overweight (BMI = 28.0 ± 0.5 kg/m^2^); and seven obese (BMI = 35.0 ± 0.5 kg/m^2^) individuals. The non‐diabetic group comprised of five lean (BMI = 22.7 ± 1.3 kg/m^2^); five overweight (BMI = 28.3 ± 0.6 kg/m^2^); and five obese (BMI = 32.5 ± 1.3 kg/m^2^) individuals. T2D patients had hypertension and hyperlipidemia, four each, while one non‐diabetic individual had hypertension. The clinico‐demographic data of the study participants are summarized in Table 1.

**Table 1:** Patients’ clinico‐demographic characteristics

	Type‐2 diabetic			Non‐diabetic		
Parameter	Lean	Overweight	Obese	Lean	Overweight	Obese
Total number (N)	1	7	7	5	5	5
Male (N)	1	3	4	0	3	1
Female (N)	0	4	3	5	2	4
Age (Yrs.)	54	45–59	46–64	28–51	33–60	24–53
Body mass index (kg/m^2^)	23.54	28 ± 0.5	35 ± 0.5	22.7 ± 1.3	28.3 ± 0.6	32.5 ± 1.3
Body fat (%)	22.5	33.5 ± 2.2	41 ± 2.4	30.9 ± 2.8	32.4 ± 1.9	39.3 ± 1.9
Fasting plasma glucose (mmol/L)	13.20	7.3 ± 0.7	8.8 ± 0.6	4.7 ± 0.2	5.5 ± 0.4	5.5 ± 0.2
Fasting plasma insulin (mIU/L)	–	10.4 ± 1.7	32 ± 9.6	4.9 ± 0.3	8.7 ± 2.9	9.6 ± 2.4
Glycated hemoglobin (HbA1c) (%)	11.20	7.4 ± 0.7	8.3 ± 0.5	5.8 ± 0.3	5.4 ± 0.3	5.5 ± 0.3
Total cholesterol (mmol/L)	5.80	4.8 ± 0.8	4.8 ± 0.3	5.3 ± 0.6	5.2 ± 0.2	5.1 ± 0.4
High‐density lipoprotein (mmol/L)	0.88	1.0 ± 0.1	1.2 ± 0.1	1.9 ± 0.2	1.3 ±0.2	1.3 ± 0.2
Low‐density lipoprotein (mmol/L)	4.00	2.9 ± 0.6	3.1 ± 0.3	3.1 ± 0.5	3.4 ± 0.3	3.3 ± 0.3
Triglycerides (mmol/L)	1.92	1.9 ± 0.4	2.0 ± 0.9	0.6 ± 0.1	1.2 ± 0.4	1.1 ± 0.2
Hypertension (N)	0	2	2	0	1	0
Hyperlipidemia (N)	0	2	2	0	0	0
Therapy	Glucophage	Zocor, lipitor glucophage metformin amaryl	Glucophage tenormin amaryl lipitor zocor	None	Diovan	None

### Ethics statement

Study protocol (RA‐2010‐003) was approved by the institutional ethics committee (Ethical Review Committee of Dasman Diabetes Institute, Kuwait). Written informed consent was obtained from all the participants for inclusion in this study.

### Anthropometric and physio‐clinical measurements

Height and weight were measured using calibrated portable electronic weighing scales and portable inflexible height measuring bars; the waist circumference was measured using constant tension tape. The whole body composition including body fat percentage, soft lean mass, and total body water were measured using IOI353 Body Composition Analyzer (Jawon Medical, South Korea). Blood pressure was measured by using Omron HEM‐907XL digital automatic sphygmomanometer (Omron Healthcare Inc. Lake Forest, CA, USA). BMI was calculated by using standard formula as follows: BMI = body weight (kg)/height (m^2^).

Peripheral blood was collected from overnight‐fasted individuals and analyzed for fasting glucose, glycated hemoglobin (HbA1c), fasting insulin, and lipid profile. Glucose and lipid profiles were measured using Siemens dimension RXL chemistry analyzer (Diamond Diagnostics, Holliston, MA). HbA1c was measured by using Variant™ device (BioRad, Hercules, CA). Plasma triglycerides were also measured using commercial kit (Chema Diagnostica, Monsano, Italy). All assays were carried out following instructions and guidelines from the manufacturers. HOMA‐IR was calculated by using standard formula as follows: HOMA‐IR = [Glucose × Insulin]/22.5 whereas, fasting plasma glucose was measured in molar units (mmol/L) and fasting insulin was measured as mIU/L.

### Collection of subcutaneous adipose tissue samples

Human adipose tissue samples (∼0.5 g) were collected by abdominal subcutaneous fat pad biopsy lateral to the umbilicus using standard surgical method. The biopsy tissue was further incised into small pieces, rinsed in cold phosphate buffered saline (PBS), fixed in 4% paraformaldehyde for 24 h and embedded in paraffin. At the same time, freshly collected adipose tissue samples (∼50–100 mg) were preserved in RNAlater or embedded in optimal cutting temperature (OCT) and stored at −80°C until use.

### Real‐time reverse‐transcription polymerase chain reaction (RT‐PCR)

Total cellular RNA was purified using RNeasy kit (Qiagen, Valencia, CA) as per manufacturer's instructions. RNA samples (1 µg each) were reverse transcribed to yield cDNA using random hexamer primers and TaqMan reverse transcription reagents (High Capacity cDNA Reverse Transcription kit; Applied Biosystems, Foster City, CA, USA). For real‐time RT‐PCR, cDNA (50 ng) was amplified using TaqMan^®^ Gene Expression MasterMix (Applied Biosystems) and gene‐specific 20× TaqMan Gene Expression Assays. The following primers (Applied Biosystems) were used in 40‐cycle PCR reaction: (IL‐18R) Hs00175381_m1; (IL‐18) Hs01038788_m1; (CD11c) Hs00174217_m1; (CD86) Hs01567026_m1; (CD68) Hs02836816_g1; (CD163) Hs00174705_m1; (TNF‐α) Hs01113624_g1; (CCL‐5) Hs00982282_m1; and (GAPDH) Hs03929097_g1, along with a target‐specific TaqMan^®^ minor groove binder (MGB) probe labeled with 6‐fluorescein amidite (FAM) dye at the 5′ end and non‐fluorescent quencher (NFQ)‐MGB at the 3′ end of the probe and using a 7500 Fast Real‐Time PCR System (Applied Biosystems). Each cycle comprised of denaturation for 15 sec at 95°C, annealing/extension for 1 min at 60°C which started after uracil DNA glycosylase (UDG) activation (50°C for 2 min) and AmpliTaq Gold (Abcam, Cambridge, MA, USA) enzyme activation (95°C for 10 min). GAPDH expression was used for internal control to normalize the differences in individual samples and expression levels of test genes relative to control gene (lean adipose tissue) were calculated using −2^ΔΔCt^ method. Relative mRNA expression was measured as fold change over average of control gene expression taken as one and data were expressed as mean ± SEM values.

### Immunohistochemistry

Paraffin‐embedded sections of subcutaneous adipose tissue (4 μm) were deparaffinized in xylene and rehydrated through descending grades of ethanol (100%, 95%, and 75%) to water. Antigen was retrieved by using target retrieval solution (pH6.0; Dako, Glostrup, Denmark) under pressure cooker boiling for 8 min followed by cooling for 15 min. After PBS washing, endogenous peroxidase activity was blocked with 3% H_2_O_2_ for 30 min and non‐specific antibody binding was blocked by treating with 5% nonfat milk for 1 h, followed by 1% bovine serum albumin solution for 1 h. Samples were incubated at room temperature overnight with isotype control rabbit IgG antibody (Millipore PP644, Merck KGaA, Cambridge, MA, USA) as well as primary antibodies against human IL‐18R1 (1:100 dilution, Abcam^®^ ab124458) and IL‐18 (1:200 dilution, Abcam^®^ ab191152). After washing, samples were incubated for 1 h with secondary HRP‐conjugated antibody and color was developed with 3,3′‐diaminobenzidine (DAB) chromogenic substrate. Specimens were washed in running tap water, lightly counterstained with Harris hematoxylin, dehydrated through ascending grades of ethanol (75%, 95%, and 100%), cleared in xylene, and finally mounted in dibutyl phthalate xylene (DPX). For protein expression quantification and data analysis, digital photomicrographs of entire sections (100×, PannoramicScan, 3DHISTECH, Budapest, Hungary) were used to quantify staining in the three selected regions outlined using Aperio ImageScope software (Aperio Vista, CA) and the regional heterogeneity was assessed in samples. Aperio‐positive pixel count algorithm version 9 was used to quantify staining intensity in tissue samples.

### Confocal microscopy

Formalin‐fixed, paraffin‐embedded adipose tissue samples were stained for immunofluorescent microscopy. After antigen retrieval and blocking as described before, co‐localization staining of samples was performed by incubating at room temperature for 1 h with adipocyte marker antibody (mouse anti‐human adiponectin 1:400 diluted antibody, Abcam^®^ ab22554) followed by overnight incubation with primary antibodies (rabbit anti‐human 1:100 diluted IL‐18R1 antibody, Abcam^®^ ab124458 and rabbit anti‐human 1:200 diluted IL‐18 antibody, Abcam^®^ ab191152) and isotype control (rabbit IgG antibody, Millipore PP644). Following washing twice with PBS‐0.05% Tween, IL‐18R1 slides were incubated for 1 h with secondary antibody (1:400 diluted goat anti‐mouse Alexa Fluor 647‐conjugated antibody, Abcam^®^ ab150115, washed three times and then incubated with goat anti‐rabbit Alexa Fluor 488 ab150077. Similarly, IL18 samples were stained with secondary antibodies (1:400 diluted goat anti‐mouse Alexa Fluor 488‐conjugated antibody, Abcam^®^ ab150113 and 1:400 diluted donkey anti‐rabbit Alexa Fluor 647‐conjugated antibody, Abcam^®^ ab150075) and washed at least thrice with PBS. The samples were counterstained with 4′,6‐diamidino‐2‐phenylindole (DAPI) (Vectashield, Vector Laboratories, H1500, Burlingame, CA, USA) and mounted with cover slips. For image processing and analysis, confocal images of adipose tissue were collected on inverted Zeiss LSM710 Spectral confocal microscope (Carl Zeiss, Gottingen, Germany) using EC Plan‐Neofluar 40×/1.30 oil DIC M27 objective lens. Samples were excited using a 488 nm diode‐pumped solid‐state laser and the 405 nm line of an argon ion laser. After laser excitation of samples, optimized emission detection bandwidths were configured by using Zeiss Zen 2010 control software.

### Statistical analysis

The data (mean ± SEM) were analyzed using GraphPad Prism software version 7.0 (La Jolla, CA). Unpaired Student *t*‐test was used to compare means between groups and Pearson's correlation (*r*) was used to assess dependence between variables. All *P* values ≤0.05 were considered as significant.

## Results

### Increased adipose tissue expression of IL‐18R in obesity

**Figure 1:**
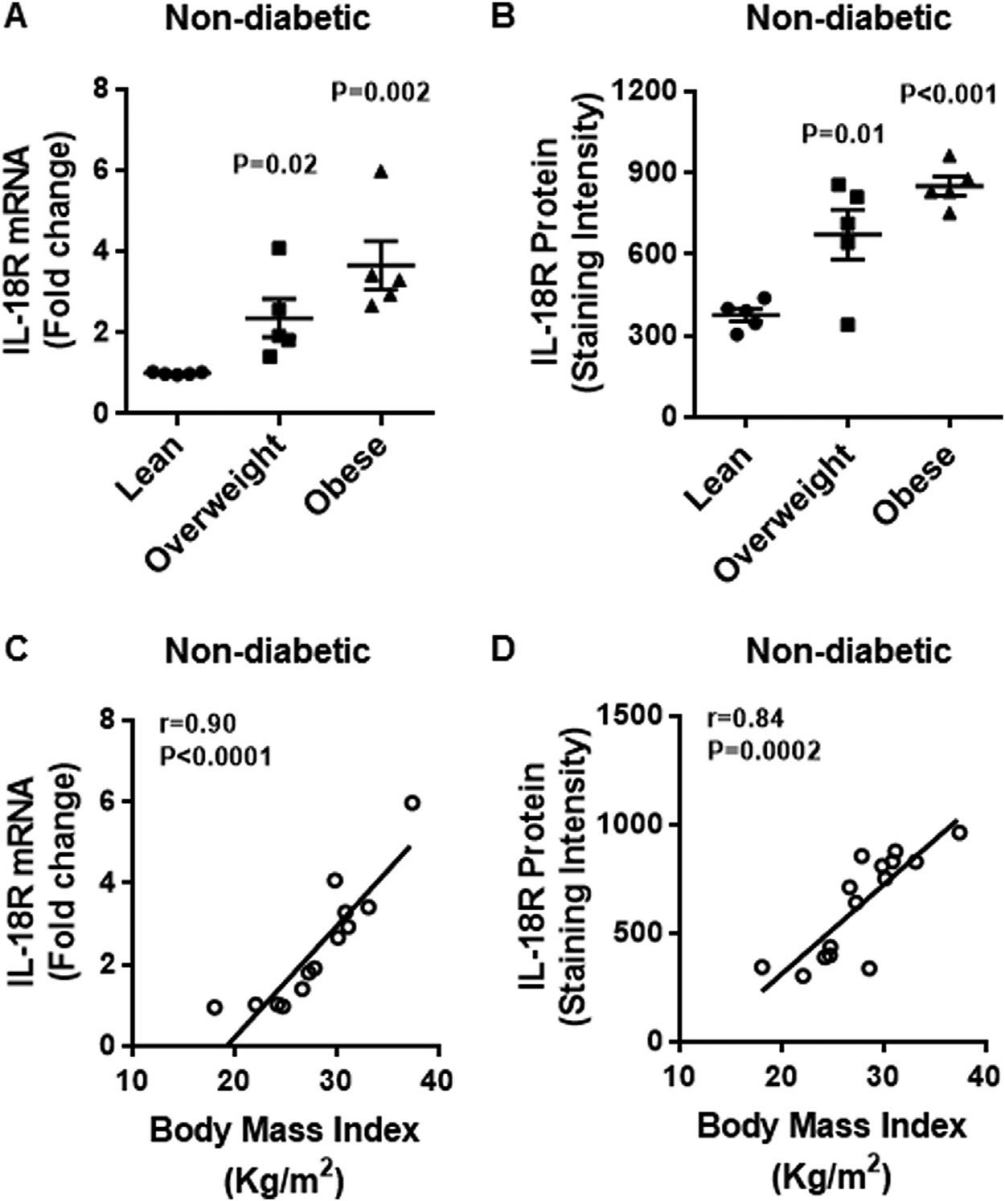
Increased adipose tissue expression of IL‐18R mRNA and protein in non‐diabetic obese and overweight individuals correlates with BMI. The adipose tissue expression of IL‐18R mRNA and protein was determined in 15 non‐diabetic individuals as described in Materials and Methods section. The increase in the IL‐18R mRNA expression detected by qRT‐PCR was represented as fold change over controls (taken as one) while the IL‐18R protein expression detected by immunohistochemistry was represented as staining intensity based on Aperio‐positive pixel counts (Aperio software algorithm version 9.0). The data (mean ± SEM) show: (A) increased expression of IL‐18R mRNA in obese (*P* = 0.002) and overweight (*P* = 0.02) individuals as compared with lean controls; and (B) increased expression of IL‐18R protein in obese (*P* < 0.001) and overweight (*P* = 0.01) as compared with lean subjects. Body mass index (BMI) correlated positively with: (C) IL‐18R mRNA expression (*r* = 0.90 *P* < 0.0001); and (D) IL‐18R protein expression (*r* = 0.84 *P* = 0.0002).

**Figure 2:**
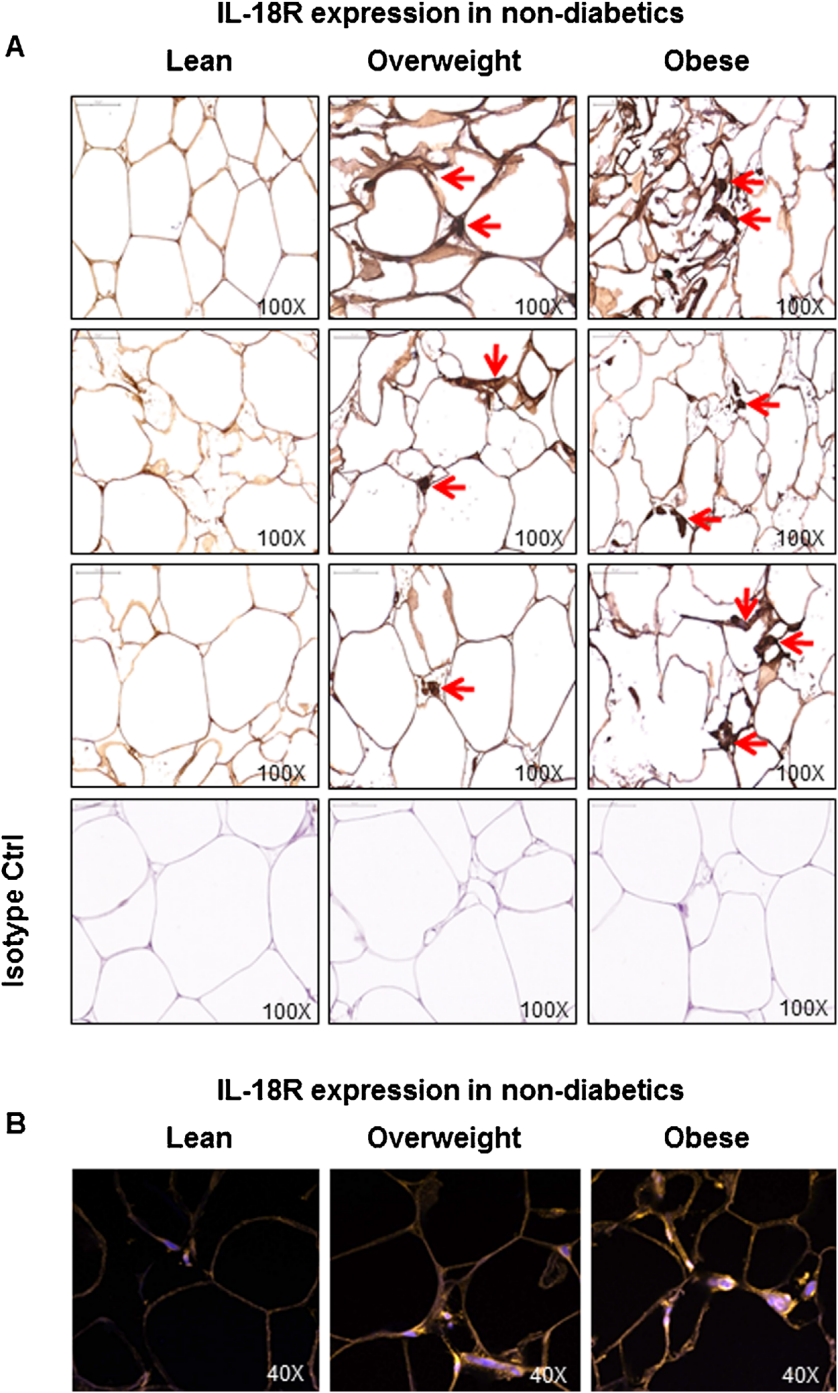
IL‐18R protein expression in the adipose tissue samples from non‐diabetic lean, overweight, and obese individuals. IL‐18R protein expression in the adipose tissue samples from non‐diabetic lean, overweight, and obese individuals was determined by immunohistochemistry and further confirmed by confocal microscopy. The representative microscopy images from three independent determinations are shown regarding: (A) immunohistochemistry (three donors per group); and (B) confocal microscopy (one donor per group). The arrow heads in immunohistochemistry images point to cells in the adipose tissue with intense staining for IL‐18R protein expression while orange staining in confocal microscopy images represents IL‐18R‐positive tissue staining.

To test the hypothesis whether the IL‐18R expression was upregulated in the adipose tissue of obese non‐diabetic/T2D individuals, we determined the expression of IL‐18R at both mRNA and protein levels in lean, overweight and obese adipose tissue samples from non‐diabetic and diabetic subjects. The data show that in non‐diabetic subjects, IL‐18R adipose tissue mRNA expression was significantly upregulated in obese and overweight as compared with lean individuals (Obese: 3.7 ± 0.6 Overweight: 2.4 ± 0.5 Lean: 1.0 ± 0.02; *P* < 0.05) (Fig. 1A). Similarly, IL‐18R protein expression in the adipose tissue was also significantly elevated in obese and overweight as compared with lean individuals (Obese: 852 ± 35 Overweight: 673 ± 91 Lean: 377 ± 23; *P* < 0.05) (Fig. 1B). The BMI correlated positively with both IL‐18R mRNA (*r* = 0.90 *P* < 0.0001) (Fig. 1C) and IL‐18R protein (*r* = 0.84 *P* = 0.0002) (Fig. 1D) expression. The representative immunohistochemistry images from three independent determinations depicting IL‐18R expression in the adipose tissue samples from non‐diabetic lean, overweight, and obese individual, three donors each, are shown in Figure 2A. The changes in IL‐18R protein expression in the adipose tissue were further confirmed by confocal microscopy as shown in Figure 2B.

**Figure 3:**
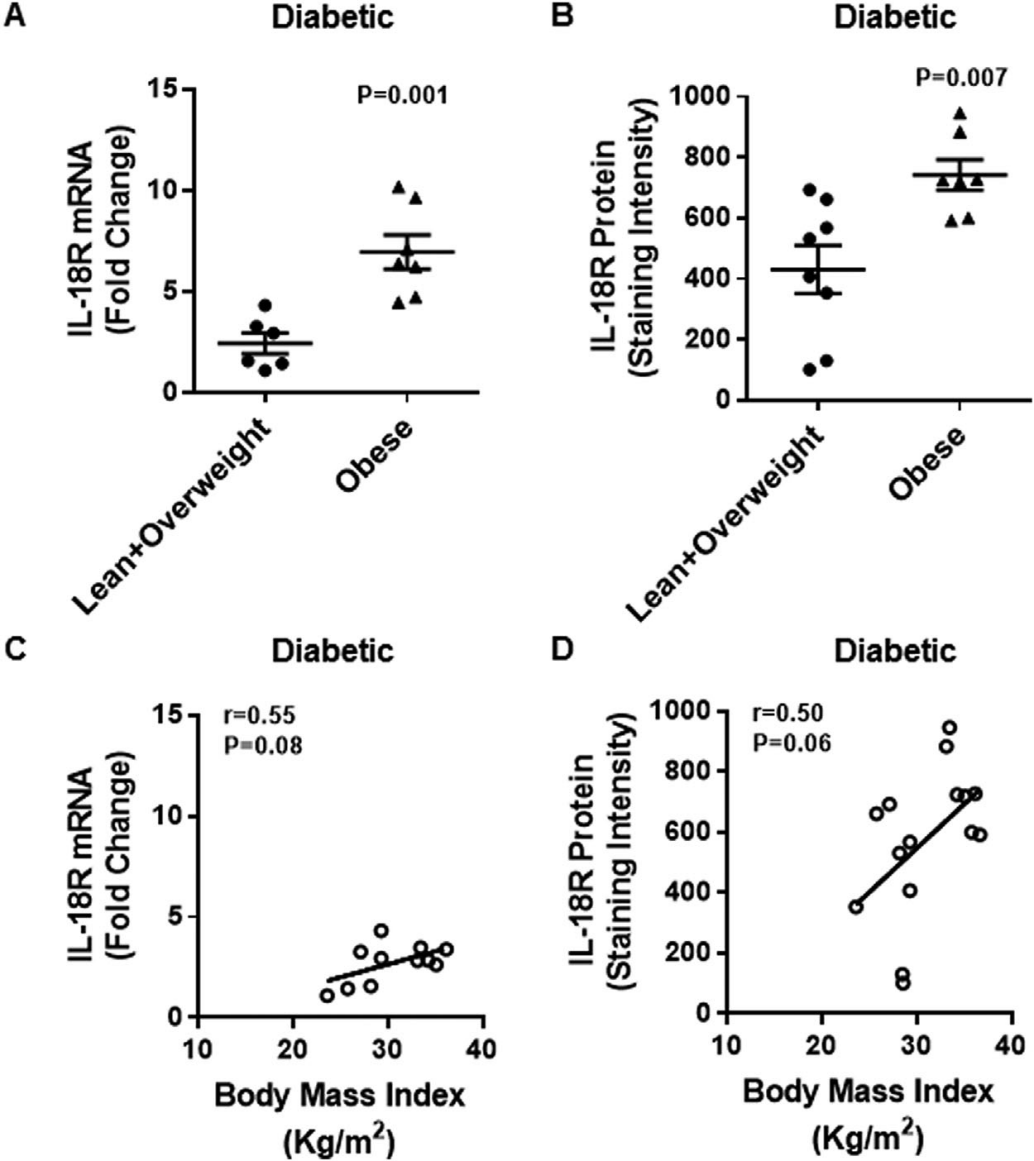
Elevated adipose tissue expression of IL‐18R mRNA and protein in type‐2 diabetic obese individuals correlates with BMI. The adipose tissue expression of IL‐18R mRNA and protein was determined in 15 type‐2 diabetic individuals. The increase in IL‐18R mRNA expression detected by qRT‐PCR was represented as fold change over control expression taken as one. The IL‐18R protein expression detected by immunohistochemistry was represented as staining intensity which was calculated using Aperio‐positive pixel counts and Aperio software algorithm (version 9.0). The data (mean ± SEM) show: (A) upregulated IL‐18R mRNA expression in obese as compared with lean/overweight individuals (*P* = 0.001); (B) increased IL‐18R protein expression in obese as compared with lean/overweight subjects (*P* = 0.007). The correlation of body mass index (BMI) with: (C) IL‐18R mRNA (*r* = 0.55 *P* = 0.08); and (D) IL‐18R protein (*r* = 0.50 *P* = 0.06) expression in the adipose tissue did not, however, reach statistical significance.

**Figure 4:**
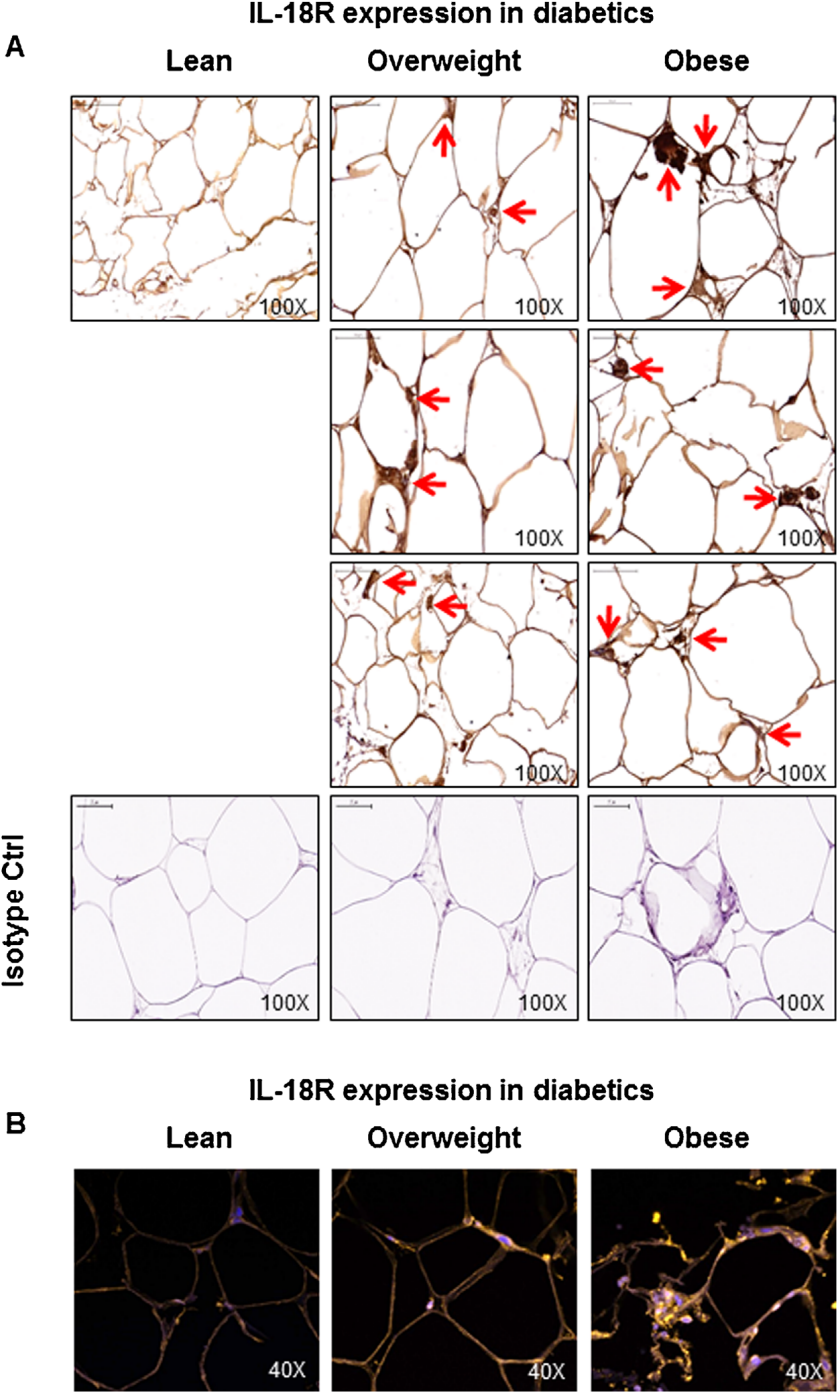
IL‐18R protein expression in the adipose tissue samples from type‐2 diabetic lean, overweight, and obese individuals. The expression of IL‐18R protein in the adipose tissue samples from type‐2 diabetic lean, overweight, and obese individuals was determined by immunohistochemistry and further confirmed by confocal microscopy. The representative images from three independent determinations are shown regarding: (A) immunohistochemistry (one donor for lean and three donors, each, for overweight and obese); and (B) confocal microscopy (one donor per group). The arrow heads in immunohistochemistry images point to cells in the adipose tissue showing intense staining for IL‐18R protein expression while orange staining in confocal microscopy images represents IL‐18R‐positive tissue staining.

As expected, in T2D patients as well, IL‐18R adipose tissue mRNA expression was significantly upregulated in obese as compared with lean/overweight individuals (Obese: 7.0 ± 0.8 Lean/Overweight: 2.4 ± 0.5; *P* < 0.05) (Fig. 3A). Similarly, IL‐18R protein expression was also elevated in obese as compared with lean/overweight individuals (Obese: 742 ± 50 Lean/Overweight: 431 ± 79; *P* < 0.05) (Fig. 3B). The BMI tended to associate positively with IL‐18R mRNA (*r* = 0.55 *P* = 0.08) (Fig. 3C) and IL‐18R protein (*r* = 0.50 *P* = 0.06) (Fig. 3D) expression in the adipose tissue. The representative immunohistochemistry images from three independent determinations of IL‐18R expression in the adipose tissue samples from diabetic lean (one donor), overweight (three donors), and obese (three donors) are shown in Figure 4A. The changes in IL‐18R protein expression in the adipose tissue samples from T2D patients were further confirmed by confocal microscopy as shown in Figure 4B.

### Elevated adipose tissue expression of cognate ligand IL‐18 in obesity

**Figure 5:**
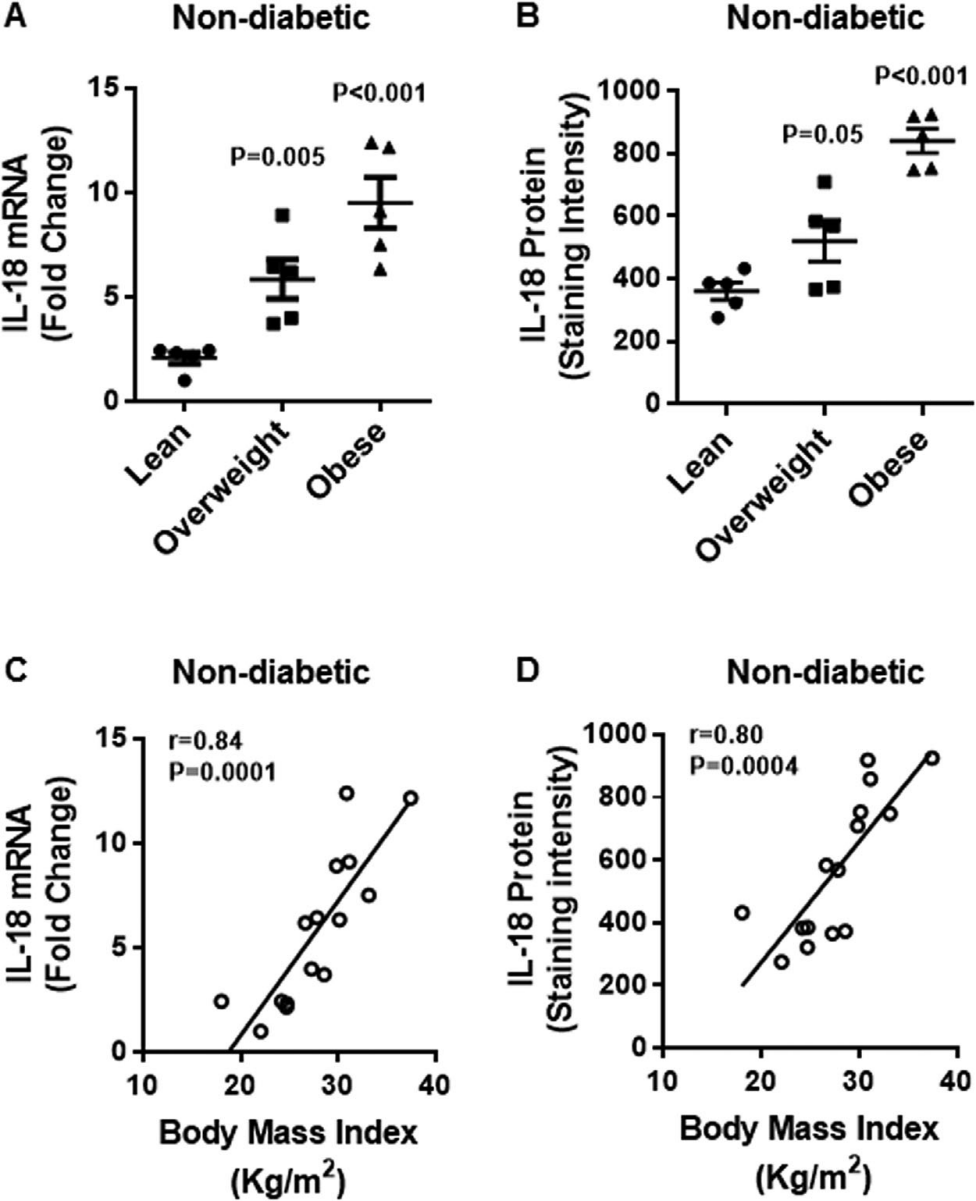
Increased adipose tissue expression of IL‐18 mRNA and protein in non‐diabetic obese and overweight individuals correlates with BMI. The adipose tissue expression of IL‐18 mRNA and protein was determined in 15 non‐diabetic individuals as described in Materials and Methods section. The increase in IL‐18 mRNA expression detected by qRT‐PCR was represented as fold change over controls taken as one while the IL‐18 protein expression detected by immunohistochemistry was represented as staining intensity based on Aperio‐positive pixel counts (Aperio software algorithm version 9.0). The data (mean ± SEM) show: (A) increased expression of IL‐18 mRNA in obese (*P* < 0.001) and overweight (*P* = 0.005) individuals as compared with lean controls; and (B) increased expression of IL‐18 protein in obese (*P* < 0.001) and overweight (*P* = 0.05) as compared with lean subjects. Body mass index (BMI) correlated positively with: (C) IL‐18 mRNA (*r* = 0.84 *P* = 0.0001); and (D) IL‐18 protein (*r* = 0.80 *P* = 0.0004) expression in the adipose tissue.

**Figure 6:**
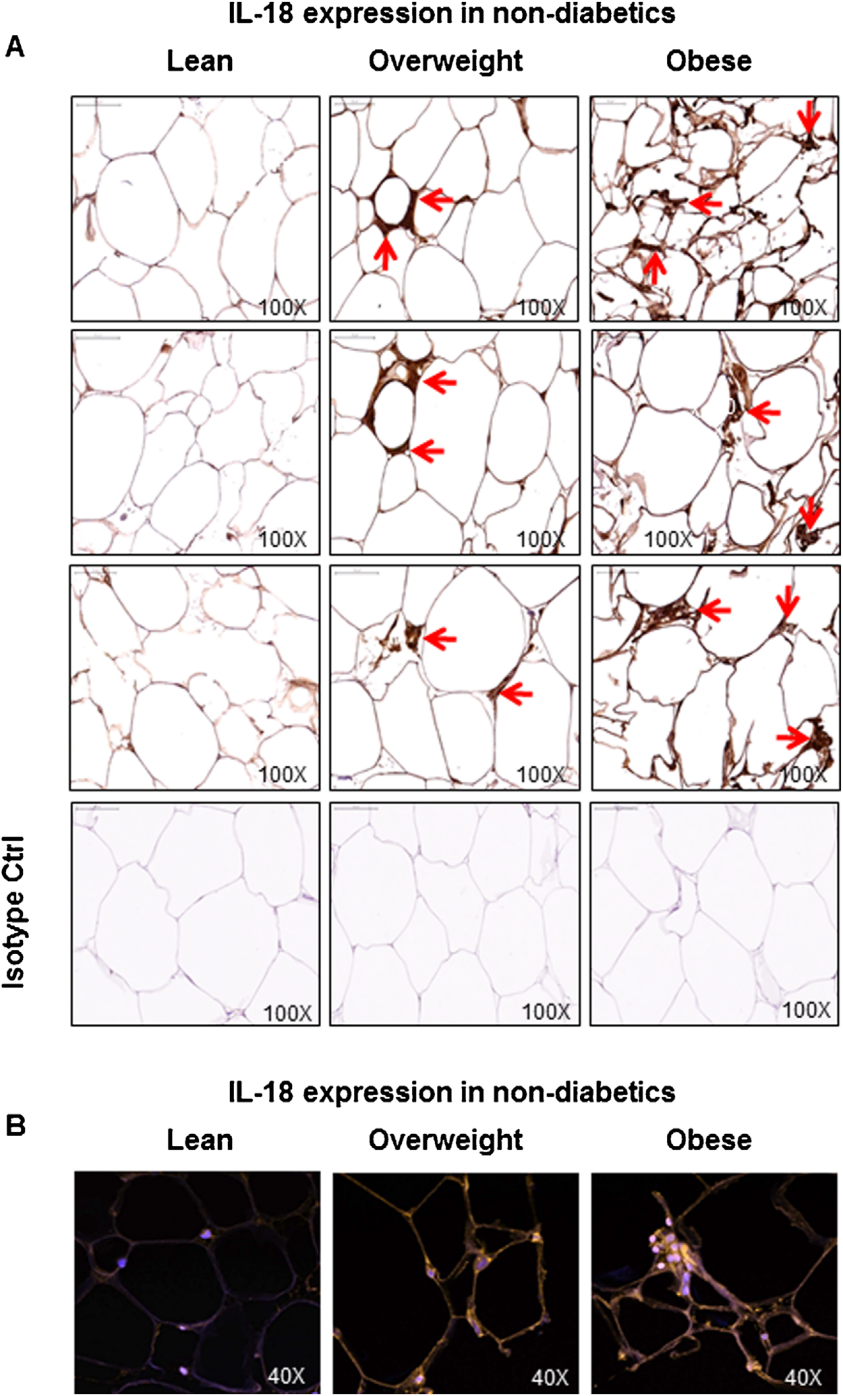
IL‐18 protein expression in the adipose tissue samples from non‐diabetic lean, overweight, and obese individuals. IL‐18 protein expression in the adipose tissue samples from non‐diabetic lean, overweight, and obese individuals was determined by immunohistochemistry and further confirmed by confocal microscopy. The representative images from three independent determinations are shown regarding: (A) immunohistochemistry (three donors per group); and (B) confocal microscopy (one donor per group). The arrow heads in immunohistochemistry images point to cells in the adipose tissue with intense staining for IL‐18 protein expression while orange staining in confocal microscopy images represents IL‐18‐positive tissue staining.

We further asked if the adipose tissue expression of the cognate ligand IL‐18 was also increased in obese diabetic/non‐diabetic subjects. The data show that in non‐diabetic subjects, the adipose tissue IL‐18 mRNA expression was significantly upregulated in obese and overweight subjects as compared with lean controls (Obese: 9.5 ± 1.2 Overweight: 5.9 ± 0.9 Lean: 2.1 ± 0.3; *P* < 0.05) (Fig. 5A). Likewise, IL‐18 protein expression in the adipose tissue was also significantly elevated in obese and overweight as compared with lean individuals (Obese: 841 ± 38 Overweight: 520 ± 66 Lean: 360 ± 27; *P* < 0.05) (Fig. 5B). The BMI correlated positively with both IL‐18 mRNA (*r* = 0.84 *P* = 0.0001) (Fig. 5C) and IL‐18 protein (*r* = 0.80 *P* = 0.0004) (Fig. 5D) expression. The representative immunohistochemistry images from three independent determinations of IL‐18 expression in the adipose tissue samples from non‐diabetic lean, overweight, and obese individuals, three each, are shown in Figure 6A. The changes in IL‐18 protein expression in the adipose tissue were also confirmed by confocal microscopy as shown in Figure 6B.

**Figure 7:**
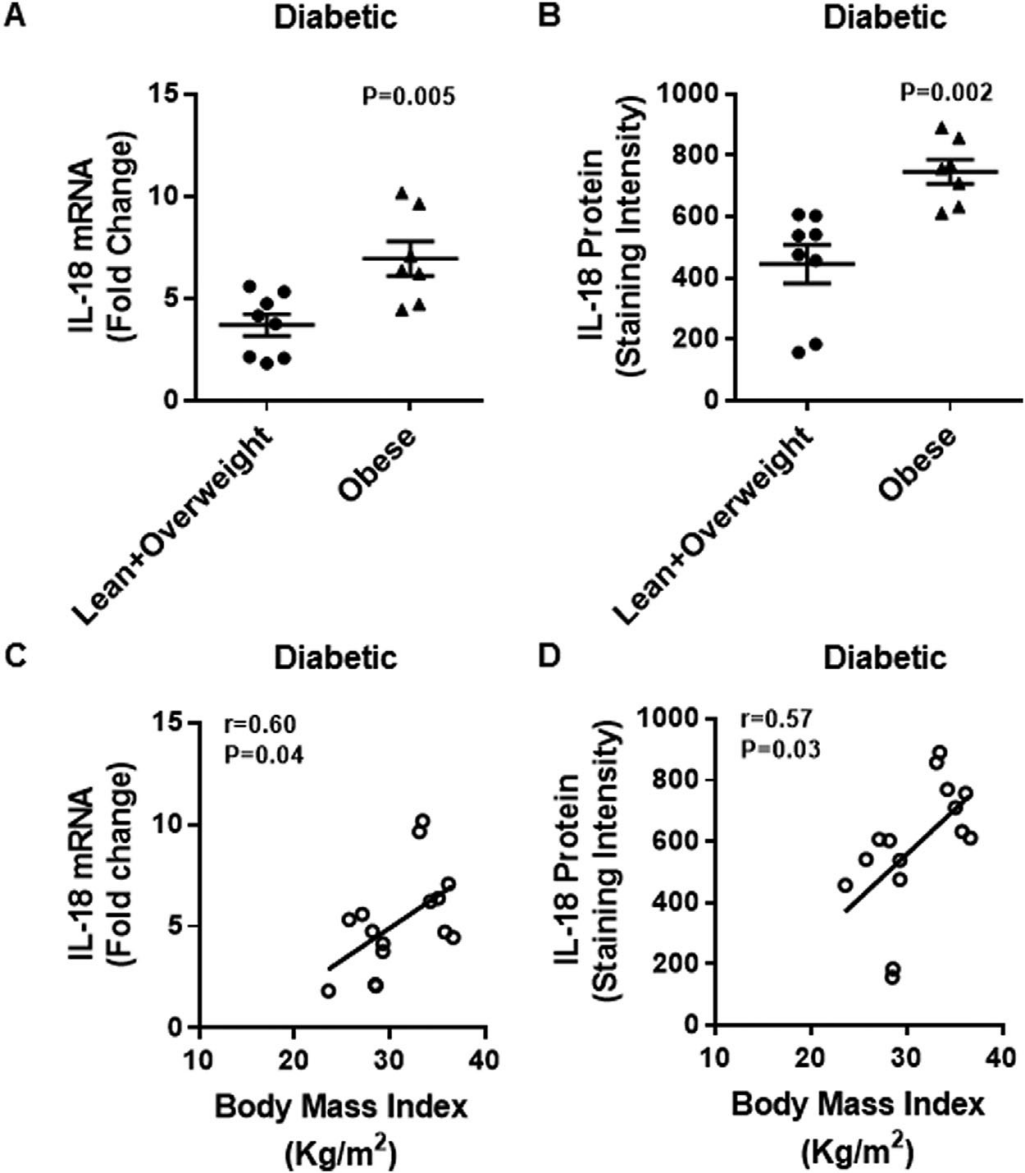
Elevated adipose tissue expression of IL‐18 mRNA and protein in type‐2 diabetic obese individuals correlates with BMI. The adipose tissue expression of IL‐18 mRNA and protein was determined in 15 type‐2 diabetic individuals. The increase in IL‐18 mRNA expression detected by qRT‐PCR was represented as fold change over control expression taken as one. IL‐18R protein expression detected by immunohistochemistry was represented as staining intensity which was calculated by using Aperio‐positive pixel counts and Aperio software algorithm (version 9.0). The data (mean ± SEM) show: (A) upregulated expression of IL‐18 mRNA in obese individuals as compared with lean/overweight individuals (*P* = 0.005); and (B) increased expression of IL‐18R protein in obese as compared with lean/overweight subjects (*P* = 0.002). Body mass index (BMI) showed a positive association with: (C) IL‐18 mRNA (*r* = 0.60 *P* = 0.04); and (D) IL‐18 protein (*r* = 0.57 *P* = 0.03) expression in the adipose tissue.

**Figure 8:**
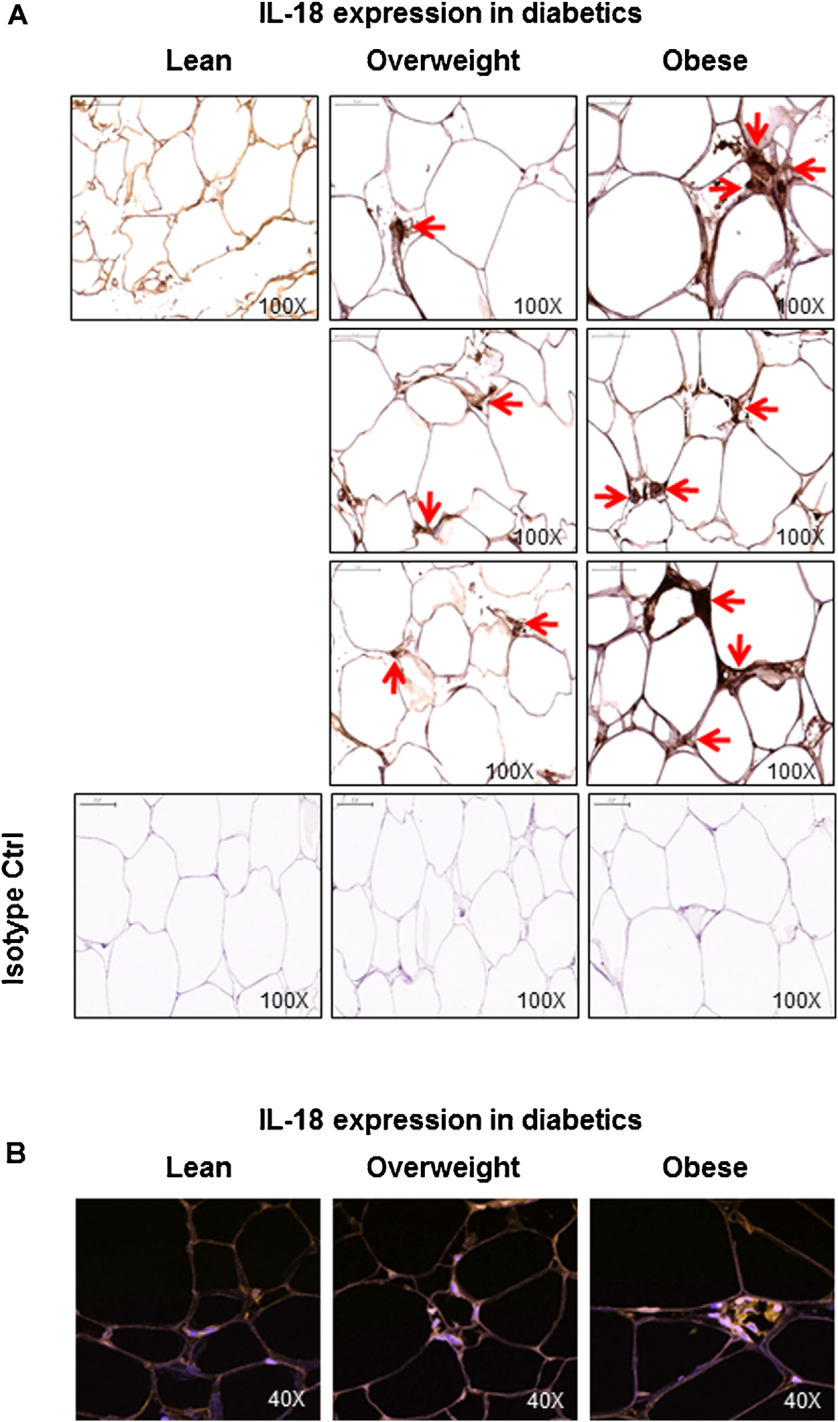
IL‐18 protein expression in the adipose tissue samples from type‐2 diabetic lean, overweight, and obese individuals. IL‐18 protein expression in the adipose tissue samples from type‐2 diabetic lean, overweight, and obese individuals was determined by immunohistochemistry and further confirmed by confocal microscopy. The representative microscopy images from three independent determinations are shown regarding: (A) immunohistochemistry (one donor for lean and three donors, each, for overweight and obese); and (B) confocal microscopy (one donor per group). The arrow heads in immunohistochemistry images point to cells in the adipose tissue showing intense staining for IL‐18 protein expression while orange staining in confocal microscopy images represents IL‐18‐positive tissue staining.

Similarly, in T2D patients, IL‐18 adipose tissue mRNA expression was significantly upregulated in obese as compared with lean/overweight individuals (Obese: 6.9 ± 0.8 Lean/Overweight: 3.7 ± 0.5; *P* < 0.05) (Fig. 7A). IL‐18 protein expression in the adipose tissue was also significantly increased in obese as compared with lean/overweight individuals (Obese: 747 ± 40 Lean/Overweight: 446 ± 63; *P* < 0.05) (Fig. 7B). Moreover, BMI correlated positively with both IL‐18 mRNA (*r* = 0.60 *P* = 0.04) (Fig. 7C) and IL‐18 protein (*r* = 0.57 *P* = 0.03) (Fig. 7D) expression. The representative immunohistochemistry images from three independent determinations of IL‐18 expression in the adipose tissue samples from diabetic lean (one donor), overweight (three donors), and obese (three donors) are shown in Figure 8A. The changes in IL‐18R protein expression in the adipose tissue samples were also confirmed by confocal microscopy as shown in Figure 8B.

### IL‐18R/IL‐18 adipose tissue expression changes relate mainly to immune cells

**Figure 9:**
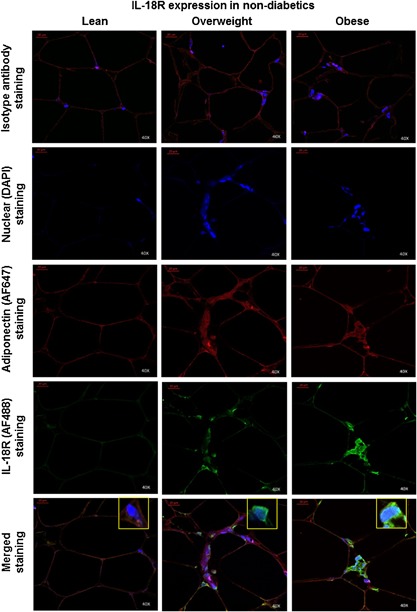
Co‐localization expression analysis of IL‐18R in the adipose tissue in obesity. Co‐localization staining for IL‐18R protein expression (green fluorescence) was performed using adiponectin as marker for adipocytes staining (red fluorescence) in the adipose tissue samples obtained from non‐diabetic lean, overweight, and obese individuals for confocal microscopy as described in Materials and Methods section. The representative microscopy images (40× magnification) from three independent determinations are shown. In the merged images, IL‐18R protein expression can be detected predominantly on immune cells known to form crown‐like structures (CLS) around the depleting adipocytes in obese adipose tissue.

**Figure 10:**
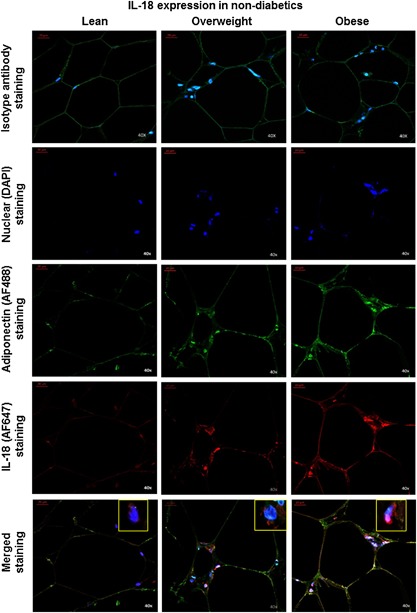
Co‐localization expression analysis of IL‐18 in the adipose tissue in obesity. Co‐localization staining for IL‐18 protein expression (red fluorescence) was performed using adiponectin as marker for adipocytes staining (green fluorescence) in the adipose tissue samples obtained from non‐diabetic lean, overweight, and obese individuals for confocal microscopy as described in Materials and Methods section. The representative microscopy images (40× magnification) from three independent determinations are shown. In the merged images, IL‐18 protein expression can be detected predominantly on immune cells known to form crown‐like structures (CLS) around the depleting adipocytes in obese adipose tissue.

Next, we wanted to know whether IL‐18R/IL‐18 expression changes in obesity were shown mainly by adipocytes or immune cells. To address this question, we performed co‐localization studies on adipose tissue samples from non‐diabetic subjects using confocal microscopy. For IL‐18R co‐localization study, we used Alexa Fluor (AF)‐488 conjugated anti‐IL‐18R antibody (green fluorescence) with AF647‐conjugated adiponectin (adipocyte marker) antibody (red fluorescence). Conversely, for IL‐18 co‐localization study, we used AF647‐conjugated anti‐IL‐18 antibody (red fluorescence) with AF488‐conjugated adiponectin marker antibody (green fluorescence). The data show that both IL‐18R (Fig. 9) and IL‐18 (Fig. 10) were expressed mainly by the immune cells around adipocytes, called as crown‐like structures (CLS; also visible in the immunohistochemistry images).

### Association of IL‐18R/IL‐18 adipose tissue expression with local inflammation and insulin resistance

We further asked whether the adipose tissue IL‐18R/IL‐18 mRNA expression in obesity and/or T2D was concordant with a local inflammatory state and insulin resistance in these individuals. To this end, we determined changes in the adipose tissue gene expression of monocyte/macrophage phenotypic (CD11c, CD86, CD68, and CD163) and cytokine/chemokine markers (TNF‐α and CCL5/RANTES) as a representative of local inflammation. HOMA‐IR index in these subjects served as a measure of insulin resistance. The data from T2D and non‐diabetic individuals, 15 each, show that the changes in IL‐18R/IL‐18 global gene expression in the adipose tissue correlated positively with macrophage phenotypic and inflammatory markers in this compartment (Table 2).

**Table 2:** Correlation of IL‐18R/IL‐18 expression with macrophage phenotype and inflammatory markers in the adipose tissue

IL‐18R/IL‐18	Markers	Pearson's correlation (r)	*P*‐value
IL‐18R	CD11c	0.56	**0.04**
	CD86	0.64	**0.01**
	CD68	0.80	**0.001**
	CD163	0.75	**0.001**
	TNF‐α	0.59	0.06
	CCL‐5/RANTES	0.57	0.08
IL‐18	CD11c	0.59	**0.02**
	CD86	0.69	**0.004**
	CD68	0.70	**0.02**
	CD163	0.78	**0.0003**
	TNF‐α	0.60	**0.04**
	CCL‐5/RANTES	0.59	**0.04**

The bold text shows significant markes.

**Figure 11:**
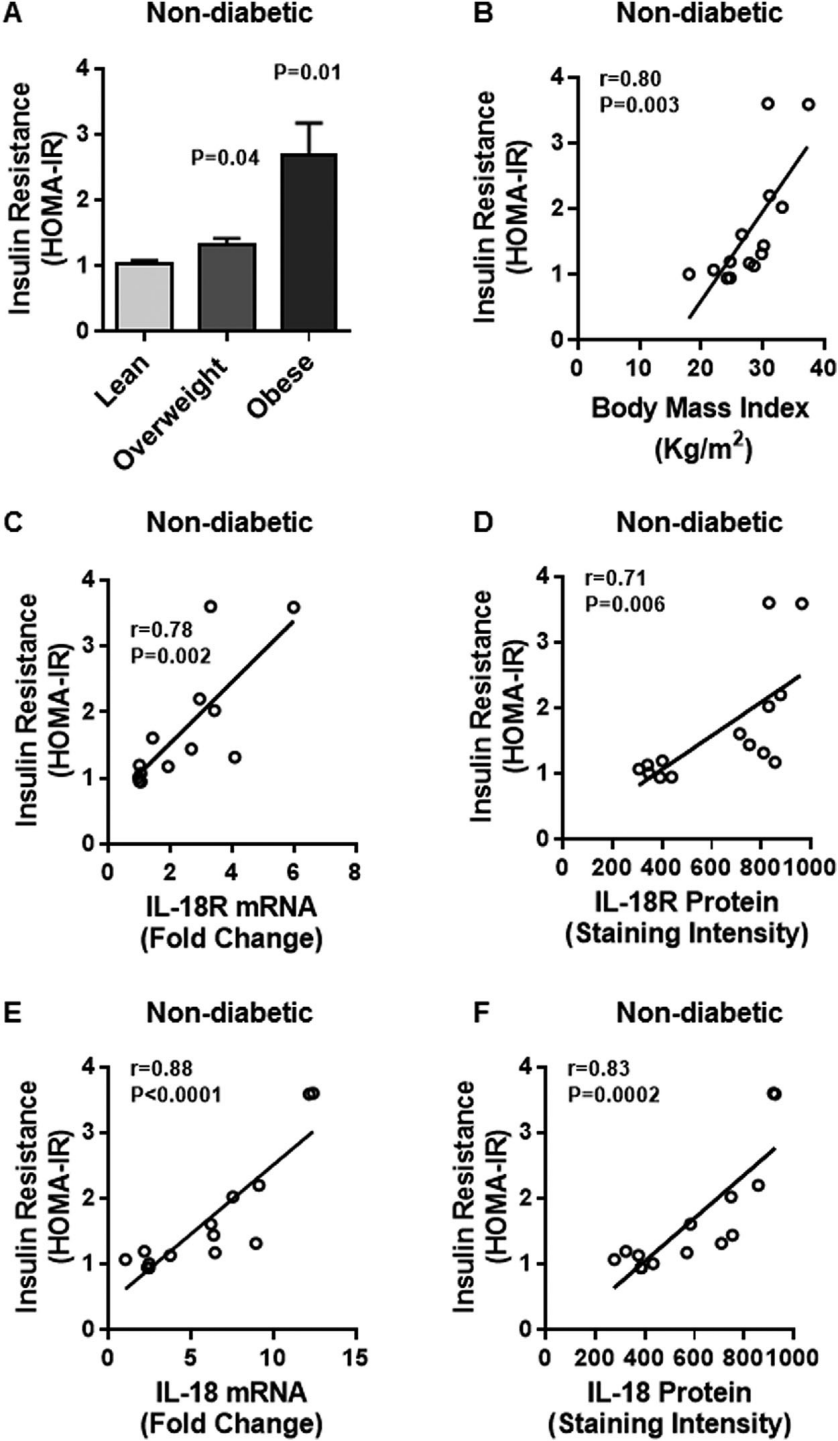
Insulin resistance profiles of non‐diabetic individuals were associated with BMI and IL‐18R/IL‐18 gene and protein expression. The insulin resistance was measured using standard formula for homeostatic model assessment of insulin resistance (HOMA‐IR) as described in Materials and Methods section. (A) HOMA‐IR (mean ± SEM) indices were significantly higher in obese (*P* = 0.01) and overweight (*P* = 0.04) individuals as compared with lean controls; and (B) Body mass index (BMI) had a positive association with HOMA‐IR (*r* = 0.80 *P* = 0.003). HOMA‐IR was also positively associated with: (C) IL‐18R mRNA (*r* = 0.78 *P* = 0.002); (D) IL‐18R protein (*r* = 0.71 *P* = 0.006); (E) IL‐18 mRNA (*r* = 0.88 *P* < 0.0001); and (F) IL‐18 protein (*r* = 0.83 *P* = 0.0002) expression in the adipose tissue.

Furthermore, in non‐diabetics, HOMA‐IR was significantly higher in obese and overweight subjects as compared with lean controls (Obese: 2.7 ± 0.5 Overweight: 1.3 ± 0.1 Lean: 1.0 ± 0.1; *P* < 0.05) (Fig. 11A). As expected, a positive correlation was found between BMI and HOMA‐IR (*r* = 0.80 *P* = 0.003) (Fig. 11B). Regarding IL‐18R global expression in the adipose tissue, HOMA‐IR correlated positively with IL‐18R mRNA (*r* = 0.78 *P* = 0.002) (Fig. 11C) and IL‐18R protein expression (*r* = 0.71 *P* = 0.006) (Fig. 11D). Similarly, HOMA‐IR also correlated positively with IL‐18 mRNA (*r* = 0.88 *P* < 0.0001) (Fig. 11E) and IL‐18 protein expression (*r* = 0.83 *P* = 0.0002) (Fig. 11F).

In T2D patients as well, HOMA‐IR was found to be significantly higher in obese than lean/overweight individuals (Obese: 12.6 ± 3.7 Lean/Overweight: 3.2 ± 0.6; *P* = 0.04) (Supplementary Fig. S1A) while the changes correlated positively with BMI (*r* = 0.63 *P* = 0.02) (Supplementary Fig. S1B). Not unexpectedly, however, HOMA‐IR in T2D patients did not correlate with the adipose tissue expression of IL‐18R mRNA (*P* = 0.13) (Supplementary Fig. S1C) and IL‐18R protein (*P* = 0.40) (Supplementary Fig. S1D) as well as IL‐18 mRNA (*P* = 0.54) (Supplementary Fig. S1E) and IL‐18 protein (*P* = 0.32) (Supplementary Fig. S1F). We further wanted to know if the changes detected in the IL‐18R and its ligand IL‐18 were mutually concordant at transcriptional and translational levels. To this effect, a good agreement was found overall between mRNA/protein expression of IL‐18R (*r* = 0.69 *P* = 0.0001) (Supplementary Fig. S2A) and IL‐18 (*r* = 0.92 *P* < 0.0001) (Supplementary Fig. S2B). Likewise, a strong concordance was also found between IL‐18R and IL‐18 at the levels of mRNA expression (*r* = 0.66 *P* = 0.0003) (Supplementary Fig. S2C) and protein expression (*r* = 0.91 *P* < 0.0001) (Supplementary Fig. S2D) in the adipose tissue.

## Discussion

In this study, for the first time to our knowledge, we show that IL‐18R mRNA/protein expression in the adipose tissue samples from diabetic/non‐diabetic obese and overweight individuals was significantly increased than their lean counterparts. Our data further show that the adipose tissue gene/protein expression of the proinflammatory cytokine IL‐18 was also significantly higher in diabetic/non‐diabetic obese and overweight subjects as compared with respective lean controls. IL‐18 is produced constitutively by many cell types including macrophages, dendritic cells, endothelial/epithelial cells, and vascular smooth muscle cells 13, 14. All these cell types, especially macrophages, are found in elevated numbers in the expanding adipose tissue in obesity/T2D and a polarization shift from the antiinflammatory M2 to the inflammatory M1 type is also observed in the adipose tissue compartment 15. IL‐18 is also produced by adipocytes 16 although, non‐fat cells are regarded as the main source of IL‐18 in the adipose tissue 17. In agreement with these data, we also found that IL‐18R/IL‐18 expression changes in obesity were manifested mainly by the immune cells (i.e., non‐fat cells) visible as CLS in the adipose tissue. IL‐18 mediates its immunobiological activities by interacting with the functional IL‐18Rα/β heterodimer receptor complex which is expressed mainly by immune cells including macrophages, dendritic cells, and T/B lymphocytes in addition to endothelial and smooth muscle cells that constitutively express the IL‐18R 13. Thus, the proinflammatory IL‐18 can stimulate all these cell types in autocrine/paracrine manner. Our data showing enhanced IL‐18R/IL‐18 global expression in the adipose tissues of diabetic/non‐diabetic obese and overweight as compared with lean subjects point to obesity as a positive modulator of the IL‐18R/IL‐18 axis in this compartment. Importantly, we also found a positive correlation between BMI and IL‐18R/IL‐18 global expression in the adipose tissue in diabetic/non‐diabetic individuals. In agreement with these data, at least in part, increased IL‐18 levels have been consistently reported in obesity, T2D, and other metabolic conditions 12, 18, 19, 20. Similarly, obesity was found to be associated with increased IL‐18 gene expression in the adipose tissue as well as elevated IL‐18 plasma levels 21. We speculate that the increased IL‐18R/IL‐18 expression in the adipose tissue in obesity/T2D may play a role to induce or augment the inflammatory responses. The emerging evidence points to a key role of chronic inflammation in the pathogenesis of metabolic diseases 22, 23, 24 and, notably, a consistent relationship was found between IL‐18 and various components of metabolic syndrome such as obesity 12, 21, insulin resistance 18, 25, lipid metabolism and dyslipidemia 20, 26, hypertension 27, 28, atherosclerosis 19, 29, cardiovascular disease 30, 31, and T2D‐associated nephropathy 32, 33.

Our data further show that HOMA‐IR was significantly higher in diabetic/non‐diabetic obese as compared with lean/overweight individuals and a positive association was found between BMI and HOMA‐IR. More importantly, the data also show that the adipose tissue expression of IL‐18R/IL‐18 in non‐diabetic individuals was associated with HOMA‐IR which is a robust clinical tool and a standard method for assessing β‐cell function and insulin resistance calculated from fasting glucose and insulin or C‐peptide concentrations. HOMA‐IR is a structural model that has steady state insulin and glucose domains constructed from the physiological dose response data of insulin production and glucose uptake, while it has also been rigorously validated against different physiological methods used for the assessment of insulin resistance 34, 35. In agreement with our data showing a correlation between increased IL‐18 gene expression in the adipose tissue in obesity and insulin resistance, Leick et al. also found that the enhanced IL‐18 mRNA expression in the adipose tissue in obesity was associated with insulin resistance 21. Dinarello et al. also showed that the elevated plasma IL‐18 levels were a marker of insulin resistance in non‐diabetic/diabetic individuals 18. Nonetheless, we did not find an association between IL‐18R/IL‐18 expression and insulin resistance in T2D patients which may be due, in part, to confounding effects of diabetes‐related immunometabolic complications or comorbidities that are often found in these subjects; and which also explains as to why HOMA‐IR is a better indicator of insulin resistance in non‐diabetic rather than diabetic subjects. A few studies reported that the changes in circulatory IL‐18 levels were associated with altered insulin sensitivity 25, 36. We did not, however, measure systemic levels of IL‐18 in the present study as it was designed to determine changes in the adipose tissue expression of IL‐18R and IL‐18 in obese/T2D individuals.

Our data represent the IL‐18R/IL‐18 axis changes with regard to obesity/T2D that are globally expressed at the gene and protein levels in the adipose tissue. To this effect, we found a strong agreement not only between gene and protein expression of IL‐18R (*r* = 0.69 *P* = 0.0001) and IL‐18 (*r* = 0.92 *P* < 0.0001) but also between IL‐18R and IL‐18 at the levels of gene (*r* = 0.66 *P* = 0.0003) and protein (*r* = 0.91 *P* < 0.0001) expression. It may thus imply that the IL‐18R/IL‐18 axis changes that are induced in obesity/T2D are mutually concordant at the transcriptional and translational levels. However, the question remains that which immunometabolic factor(s) play a role in the induction or upregulation of IL‐18R and IL‐18 in the adipose tissue in obesity and/or T2D. Interestingly, Esposito et al. demonstrated that hyperglycemia and oxidative stress were able to elevate the circulating levels of proinflammatory cytokines including TNF‐α, IL‐6, and IL‐18 37. Regarding previous studies on IL‐18R and IL‐18 expression, proinflammatory cytokines such as TNF‐α was shown to upregulate the expression of IL‐18R 38 whereas IL‐18 was reported to promote the release of TNF‐α by macrophages 39. Adipose tissue infiltrating monocytes/macrophages can play a role in the adipose tissue inflammation in morbid obesity and, in this regard, our data show that the increased adipose tissue IL‐18R/IL‐18 gene expression in obesity/T2D was associated positively with monocyte/macrophage markers such as CD11c, CD86, CD68, and CD163. The expression of IL‐18 mRNA also correlated positively with the gene expression of TNF‐α and CCL‐5/RANTES which may have pathobiological significance in metabolic inflammation. TNF‐α is a well‐known proinflammatory cytokine that is secreted mainly by activated monocytes/macrophages and is a potent regulator/mediator of inflammation, an acute phase reactant and endogenous pyrogen 40. Due to its role in metabolic inflammation and systemic insulin resistance, TNF‐α is regarded as a key component of the obesity‐diabetes link 41. CCL‐5/RANTES has been shown to cause inflammation by promoting macrophage recruitment and survival in human adipose tissue 42. Monocytes/macrophages are also known to produce CCL‐2/MCP‐1 which is one of the key chemokines that regulate monocyte/macrophage migration and infiltration into sites of inflammation 43. In our study, however, the correlation between IL‐18 and CCL‐2/MCP‐1 did not reach statistical significance. A hyperglycemic proinflammatory milieu that is consistently found in obesity/T2D can be expected to induce and promote the IL‐18R/IL‐18 expression in the adipose tissue which may have pathobiological implications in metabolic disease. In corroboration with this line of argument, Troseid et al. suggested that hyperglycemia and inflammation had a mutually potentiating effect in cardiovascular risk prediction and they also showed that IL‐18 was a strong independent predictor of cardiovascular events in metabolic syndrome patients, especially in those with elevated fasting glucose levels 31.

Overall, our results are limited by the small number of participants that could be recruited at the initial phase of this study. Besides, the present findings highlight correlation of the altered adipose tissue expression of IL‐18R/IL‐18 with tissue inflammation and insulin resistance in obesity while the systemic changes in relation to inflammatory biomarkers such as TNF‐α, IL‐1β, IFN‐γ, IL‐18, CCL‐2, CCL‐5, and C‐reactive protein still remain unclear. Therefore, further studies will be required to validate these preliminary findings as well as to identify key factor(s) that can lead to upregulation of IL‐18R/IL‐18 expression in the adipose tissue in metabolic disease.

In conclusion, our data show that IL‐18R/IL‐18 mRNA and protein expression was elevated (although mainly on immune cells) in the adipose tissue in obesity. This upregulated IL‐18R/IL‐18 gene expression in the adipose tissue was found to be associated with tissue inflammation and insulin resistance.

## Authors’ Contributions

All authors have made substantial contribution to the study and are thoroughly familiar with the original data. The contribution of each author is as follows: RA conceived and designed the study, guided experiments, performed data analysis/graphics, edited the manuscript, and procured funds. RT and SK collected and processed samples, conducted experiments, collected data, and reviewed the manuscript. SS guided experiments, carried out data analysis, interpreted results, and wrote the manuscript.

## Conflict of Interest

The authors declare that there is no conflict of interest involved.

## Supporting information

Additional supporting information may be found in the online version of this article at the publisher's web‐site.

**Figure S1**. Insulin resistance profiles of subjects with type‐2 diabetes are associated with BMI.

**Figure S2**. Concordance between mRNA/protein expression of IL‐18R and IL‐18 as well as between IL‐18/IL‐18R at gene and protein expression levels in the adipose tissue.

## References

[1] Makki, K. , P. Froguel , and I. Wolowczuk . 2013 Adipose tissue in obesity‐related inflammation and insulin resistance: cells, cytokines, and chemokines. ISRN Inflamm. 2013:139239. 24455420 10.1155/2013/139239 PMC3881510

[2] McInnes, I. B. , J. A. Gracie , B. P. Leung , X. Q. Wei , and F. Y. Liew . 2000 Interleukin 18: a pleiotropic participant in chronic inflammation. Immunol. Today 21(7):312–315. 10871869 10.1016/s0167-5699(00)01648-0

[3] Ghayur, T. , S. Banerjee , M. Hugunin , D. Butler , L. Herzog , A. Carter , L. Quintal , L. Sekut , R. Talanian , M. Paskind , et al. 1997 Caspase‐1 processes IFN‐gamma‐inducing factor and regulates LPS‐induced IFN‐gamma production. Nature 386(6625):619–623. 9121587 10.1038/386619a0

[4] Troseid, M. , I. Seljeflot , and H. Arnesen . 2010 The role of interleukin‐18 in the metabolic syndrome. Cardiovasc. Diabetol. 9:11. 20331890 10.1186/1475-2840-9-11 PMC2858122

[5] Gracie, J. A. 2004 Interleukin‐18 as a potential target in inflammatory arthritis. Clin. Exp. Immunol. 136(3):402–404. 15147340 10.1111/j.1365-2249.2004.02475.x PMC1809042

[6] Kato, Z. , J. Jee , H. Shikano , M. Mishima , I. Ohki , H. Ohnishi , A. Li , K. Hashimoto , E. Matsukuma , K. Omoya , et al. 2003 The structure and binding mode of interleukin‐18. Nat. Struct. Biol. 10(11):966–971. 14528293 10.1038/nsb993

[7] Adachi, O. , T. Kawai , K. Takeda , M. Matsumoto , H. Tsutsui , M. Sakagami , K. Nakanishi , and S. Akira . 1998 Targeted disruption of the MyD88 gene results in loss of IL‐1‐ and IL‐18‐mediated function. Immunity 9(1):143–150. 9697844 10.1016/s1074-7613(00)80596-8

[8] Opstad, T. B. , A. A. Pettersen , H. Arnesen , and I. Seljeflot . 2011 Circulating levels of IL‐18 are significantly influenced by the IL‐18 +183 A/G polymorphism in coronary artery disease patients with diabetes type 2 and the metabolic syndrome: an observational study. Cardiovasc. Diabetol. 10:110. 22141572 10.1186/1475-2840-10-110 PMC3295692

[9] Nold, M. , A. Goede , W. Eberhardt , J. Pfeilschifter , and H. Muhl . 2003 IL‐18 initiates release of matrix metalloproteinase‐9 from peripheral blood mononuclear cells without affecting tissue inhibitor of matrix metalloproteinases‐1: suppression by TNF alpha blockage and modulation by IL‐10. Naunyn Schmiedebergs Arch. Pharmacol. 367(1):68–75. 12616343 10.1007/s00210-002-0648-5

[10] Wood, I. S. , B. Wang , J. R. Jenkins , and P. Trayhurn . 2005 The pro‐inflammatory cytokine IL‐18 is expressed in human adipose tissue and strongly upregulated by TNF‐alpha in human adipocytes. Biochem. Biophys. Res. Commun. 337(2):422–429. 16188228 10.1016/j.bbrc.2005.09.068

[11] Ahmad, R. , P. K. Shihab , R. Thomas , M. Alghanim , A. Hasan , S. Sindhu , and K. Behbehani . 2015 Increased expression of the interleukin‐1 receptor‐associated kinase (IRAK)‐1 is associated with adipose tissue inflammatory state in obesity. Diabetol. Metab. Syndr. 7:71. 26312071 10.1186/s13098-015-0067-7 PMC4549832

[12] Bruun, J. M. , B. Stallknecht , J. W. Helge , and B. Richelsen . 2007 Interleukin‐18 in plasma and adipose tissue: effects of obesity, insulin resistance, and weight loss. Eur. J. Endocrinol. 157(4):465–471. 17893261 10.1530/EJE-07-0206

[13] Gerdes, N. , G. K. Sukhova , P. Libby , R. S. Reynolds , J. L. Young , and U. Schonbeck . 2002 Expression of interleukin (IL)‐18 and functional IL‐18 receptor on human vascular endothelial cells, smooth muscle cells, and macrophages: implications for atherogenesis. J. Exp. Med. 195(2):245–257. 11805151 10.1084/jem.20011022 PMC2193607

[14] Dinarello, C. A. 2007 Interleukin‐18 and the pathogenesis of inflammatory diseases. Semin. Nephrol. 27(1):98–114. 17336692 10.1016/j.semnephrol.2006.09.013

[15] Lumeng, C. N. , J. L. Bodzin , and A. R. Saltiel . 2007 Obesity induces a phenotypic switch in adipose tissue macrophage polarization. J. Clin. Invest. 117(1):175–184. 17200717 10.1172/JCI29881 PMC1716210

[16] Skurk, T. , H. Kolb , S. Muller‐Scholze , K. Rohrig , H. Hauner , and C. Herder . 2005 The proatherogenic cytokine interleukin‐18 is secreted by human adipocytes. Eur. J. Endocrinol. 152(6):863–868. 15941925 10.1530/eje.1.01897

[17] Fain, J. N. , D. S. Tichansky , and A. K. Madan . 2006 Most of the interleukin 1 receptor antagonist, cathepsin S, macrophage migration inhibitory factor, nerve growth factor, and interleukin 18 release by explants of human adipose tissue is by the non‐fat cells, not by the adipocytes. Metabolism 55(8):1113–1121. 16839849 10.1016/j.metabol.2006.04.008

[18] Dinarello, C. A. 2007 Interleukin‐18 and the pathogenesis of inflammatory diseases. Semin. Nephrol. 27(1):98–114. 17336692 10.1016/j.semnephrol.2006.09.013

[19] Zirlik, A. , S. M. Abdullah , N. Gerdes , L. MacFarlane , U. Schonbeck , A. Khera , D. K. McGuire , G. L. Vega , S. Grundy , P. Libby , et al. 2007 Interleukin‐18, the metabolic syndrome, and subclinical atherosclerosis: results from the Dallas Heart Study. Arterioscler. Thromb. Vasc. Biol. 27(9):2043–2049. 17626902 10.1161/ATVBAHA.107.149484

[20] Hung, J. , B. M. McQuillan , C. M. Chapman , P. L. Thompson , and J. P. Beilby . 2005 Elevated interleukin‐18 levels are associated with the metabolic syndrome independent of obesity and insulin resistance. Arterioscler. Thromb. Vasc. Biol. 25(6):1268–1273. 15790931 10.1161/01.ATV.0000163843.70369.12

[21] Leick, L. , B. Lindegaard , D. Stensvold , P. Plomgaard , B. Saltin , and H. Pilegaard . 2007 Adipose tissue interleukin‐18 mRNA and plasma interleukin‐18: effect of obesity and exercise. Obesity 15(2):356–363. 17299108 10.1038/oby.2007.528

[22] Donath, M. Y. , and S. E. Shoelson . 2011 Type 2 diabetes as an inflammatory disease. Nat. Rev. Immunol. 11(2):98–107. 21233852 10.1038/nri2925

[23] Mathis, D. , and S. E. Shoelson . 2011 Immunometabolism: an emerging frontier. Nat. Rev. Immunol. 11(2):81. 21469396 10.1038/nri2922 PMC4784680

[24] Esser, N. , S. Legrand‐Poels , J. Piette , A. J. Scheen , and N. Paquot . 2014 Inflammation as a link between obesity, metabolic syndrome and type 2 diabetes. Diabetes Res. Clin. Pract. 105(2):141–150. 24798950 10.1016/j.diabres.2014.04.006

[25] Straczkowski, M. , I. Kowalska , A. Nikolajuk , E. Otziomek , A. Adamska , M. Karolczuk‐Zarachowicz , and M. Gorska . 2007 Increased serum interleukin‐18 concentration is associated with hypoadiponectinemia in obesity, independently of insulin resistance. Int. J. Obesity 31(2):221–225. 10.1038/sj.ijo.0803421 16770329

[26] Yamanishi, K. , S. Maeda , S. Kuwahara‐Otani , Y. Watanabe , M. Yoshida , K. Ikubo , D. Okuzaki , Y. El‐Darawish , W. Li , K. Nakasho , et al. 2016 Interleukin‐18‐deficient mice develop dyslipidemia resulting in nonalcoholic fatty liver disease and steatohepatitis. Transl. Res. 173:101–114 e107. 27063959 10.1016/j.trsl.2016.03.010

[27] Rabkin, S. W. 2009 The role of interleukin 18 in the pathogenesis of hypertension‐induced vascular disease. Nat. Clin. Pract. Cardiovasc. Med. 6(3):192–199. 19234499 10.1038/ncpcardio1453

[28] Krishnan, S. M. , C. G. Sobey , E. Latz , A. Mansell , and G. R. Drummond . 2014 IL‐1beta and IL‐18: inflammatory markers or mediators of hypertension? Br. J. Pharmacol. 171(24):5589–5602. 25117218 10.1111/bph.12876 PMC4290704

[29] Wang, J. , C. Sun , N. Gerdes , C. Liu , M. Liao , J. Liu , M. A. Shi , A. He , Y. Zhou , G. K. Sukhova , et al. 2015 Interleukin 18 function in atherosclerosis is mediated by the interleukin 18 receptor and the Na‐Cl co‐transporter. Nat. Med. 21(7):820–826. 26099046 10.1038/nm.3890 PMC4554539

[30] Evans, J. , M. Collins , C. Jennings , L. van der Merwe , I. Soderstrom , T. Olsson , N. S. Levitt , E. V. Lambert , and J. H. Goedecke . 2007 The association of interleukin‐18 genotype and serum levels with metabolic risk factors for cardiovascular disease. Eur. J. Endocrinol. 157(5):633–640. 17984243 10.1530/EJE-07-0463

[31] Troseid, M. , I. Seljeflot , E. M. Hjerkinn , and H. Arnesen . 2009 Interleukin‐18 is a strong predictor of cardiovascular events in elderly men with the metabolic syndrome: synergistic effect of inflammation and hyperglycemia. Diabetes Care 32(3):486–492. 19092166 10.2337/dc08-1710 PMC2646034

[32] Fujita, T. , N. Ogihara , Y. Kamura , A. Satomura , Y. Fuke , C. Shimizu , Y. Wada , and K. Matsumoto . 2012 Interleukin‐18 contributes more closely to the progression of diabetic nephropathy than other diabetic complications. Acta Diabetol. 49(2):111–117. 20186552 10.1007/s00592-010-0178-4

[33] Nakamura, A. , K. Shikata , M. Hiramatsu , T. Nakatou , T. Kitamura , J. Wada , T. Itoshima , and H. Makino . 2005 Serum interleukin‐18 levels are associated with nephropathy and atherosclerosis in Japanese patients with type 2 diabetes. Diabetes Care 28(12):2890–2895. 16306550 10.2337/diacare.28.12.2890

[34] Matthews, D. R. , J. P. Hosker , A. S. Rudenski , B. A. Naylor , D. F. Treacher , and R. C. Turner . 1985 Homeostasis model assessment: insulin resistance and beta‐cell function from fasting plasma glucose and insulin concentrations in man. Diabetologia 28(7):412–419. 3899825 10.1007/BF00280883

[35] Wallace, T. M. , J. C. Levy , and D. R. Matthews . 2004 Use and abuse of HOMA modeling. Diabetes Care 27(6):1487–1495. 15161807 10.2337/diacare.27.6.1487

[36] Bosch, M. , A. Lopez‐Bermejo , J. Vendrell , M. Musri , W. Ricart , and J. M. Fernandez‐Real . 2005 Circulating IL‐18 concentration is associated with insulin sensitivity and glucose tolerance through increased fat‐free mass. Diabetologia 48(9):1841–1843. 16052331 10.1007/s00125-005-1859-3

[37] Esposito, K. , F. Nappo , R. Marfella , G. Giugliano , F. Giugliano , M. Ciotola , L. Quagliaro , A. Ceriello , and D. Giugliano . 2002 Inflammatory cytokine concentrations are acutely increased by hyperglycemia in humans: role of oxidative stress. Circulation 106(16):2067–2072. 12379575 10.1161/01.cir.0000034509.14906.ae

[38] Krasna, E. , L. Kolesar , A. Slavcev , S. Valhova , B. Kronosova , M. Jaresova , and I. Striz . 2005 IL‐18 receptor expression on epithelial cells is upregulated by TNF alpha. Inflammation 29(1):33–37. 16502344 10.1007/s10753-006-8967-1

[39] Dai, S. M. , H. Matsuno , H. Nakamura , K. Nishioka , and K. Yudoh . 2004 Interleukin‐18 enhances monocyte tumor necrosis factor alpha and interleukin‐1beta production induced by direct contact with T lymphocytes: implications in rheumatoid arthritis. Arthritis Rheum. 50(2):432–443. 14872485 10.1002/art.20064

[40] Bradley, J. R. 2008 TNF‐mediated inflammatory disease. J. Pathol. 214(2):149–160. 18161752 10.1002/path.2287

[41] Hotamisligil, G. S. , and B. M. Spiegelman . 1994 Tumor necrosis factor alpha: a key component of the obesity‐diabetes link. Diabetes 43(11):1271–1278. 7926300 10.2337/diab.43.11.1271

[42] Keophiphath, M. , C. Rouault , A. Divoux , K. Clement , and D. Lacasa . 2010 CCL5 promotes macrophage recruitment and survival in human adipose tissue. Arterioscler. Thromb. Vasc. Biol. 30(1):39–45. 19893003 10.1161/ATVBAHA.109.197442

[43] Deshmane, S. L. , S. Kremlev , S. Amini , and B. E. Sawaya . 2009 Monocyte chemoattractant protein‐1 (MCP‐1): an overview. J. Interferon Cytokine Res. 29(6):313–326. 19441883 10.1089/jir.2008.0027 PMC2755091

