# Supplementary figures and images for: Increased adipose tissue expression of IL‐18R and its ligand IL‐18 associates with inflammation and insulin resistance in obesity
Source: Immun Inflamm Dis. 2017 May 15;5(3):318–35. doi: 10.1002/iid3.170 (PMC5569378; doi:10.1002/iid3.170)

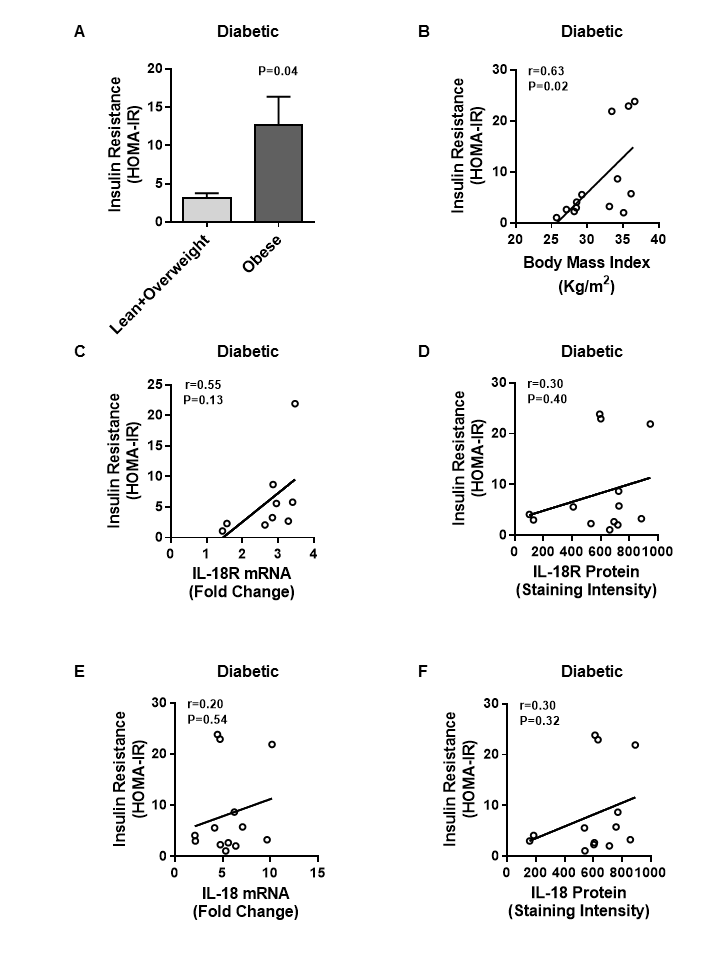

Supplement: Supplementary file 1 — Figure S1. Insulin resistance profiles of subjects with type‐2 diabetes are associated with BMI. [file IID3-5-318-s001.tif]

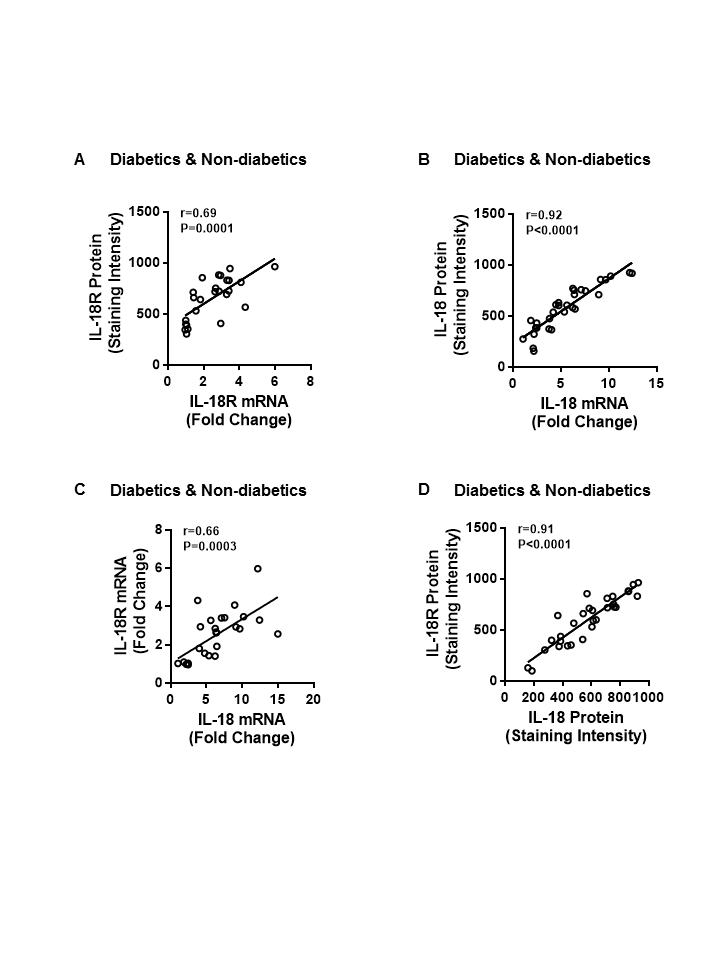

Supplement: Supplementary file 2 — Figure S2. Concordance between mRNA/protein expression of IL‐18R and IL‐18 as well as between IL‐18/IL‐18R at gene and protein expression levels in the adipose tissue. [file IID3-5-318-s002.tif]
